# Cytokine Profiles as Potential Prognostic and Therapeutic Markers in SARS-CoV-2-Induced ARDS

**DOI:** 10.3390/jcm11112951

**Published:** 2022-05-24

**Authors:** Francesco Salton, Paola Confalonieri, Giuseppina Campisciano, Rossella Cifaldi, Clara Rizzardi, Daniele Generali, Riccardo Pozzan, Stefano Tavano, Chiara Bozzi, Giulia Lapadula, Gianfranco Umberto Meduri, Marco Confalonieri, Manola Comar, Selene Lerda, Barbara Ruaro

**Affiliations:** 1 Pulmonology Unit, University Hospital of Trieste, University of Trieste, 34149 Trieste, Italy; paola.confalonieri.24@gmail.com (P.C.); rossella.cifaldi@gmail.com (R.C.); riccardo.pozzan@outlook.it (R.P.); stefano.tavano95@gmail.com (S.T.); chiara.bozzi93@gmail.com (C.B.); giulia31.lapadula@gmail.com (G.L.); mconfalonieri@units.it (M.C.); barbara.ruaro@yahoo.it (B.R.); 2 Department of Medical, Surgical and Health Sciences, University of Trieste, 34149 Trieste, Italy; crizzardi@units.it (C.R.); dgenerali@units.it (D.G.); manola.comar@burlo.trieste.it (M.C.); 3 Department of Advanced Translational Microbiology, Institute for Maternal and Child Health-IRCCS “Burlo Garofolo”, 34137 Trieste, Italy; g.campisciano@gmail.com; 4 Department of Pathology, University Hospital of Trieste, 34149 Trieste, Italy; 5 Department of Medicine, Pulmonary, Critical Care, and Sleep Medicine Division, University of Tennessee Health Science Center, Memphis, TN 38163, USA; gmeduri@uthsc.edu; 6 24ore Business School, Via Monte Rosa 91, 20149 Milano, Italy; selenelerda@gmail.com

**Keywords:** acute respiratory distress syndrome (ARDS), cytokines, glucocorticoids, COVID-19, non-invasive ventilation (NIV)

## Abstract

Background. Glucocorticoids (GCs) have been shown to reduce mortality and the need for invasive mechanical ventilation (IMV) in SARS-CoV-2-induced acute respiratory distress syndrome (ARDS). It has been suggested that serum cytokines levels are markers of disease severity in ARDS, although there is only limited evidence of a relationship between the longitudinal cytokine profile and clinical outcomes in patients with SARS-CoV-2-induced ARDS treated with GC. Methods. We conducted a single-center observational study to investigate serial plasma cytokine levels in 17 patients supported with non-invasive ventilation (NIV) in order to compare the response in five patients who progressed to IMV versus 12 patients who continued with NIV alone. All patients received methylprednisolone 80 mg/day continuous infusion until clinical improvement. Results. The study groups were comparable at baseline. All patients survived. Although IL-6 was higher in the NIV group at baseline, several cytokines were significantly higher in the IMV group on day 7 (IL-6, IL-8, IL-9, G-CSF, IP-10, MCP-1, MIP-1α) and 14 (IL-6, IL-8, IL-17, G-CSF, MIP-1α, RANTES). No significant differences were observed between groups on day 28. Conclusions. Patients in the IMV group had higher inflammation levels at intubation than the NIV group, which may indicate a higher resistance to glucocorticoids. Higher GC doses or a longer treatment duration in these patients might have allowed for a better control of inflammation and a better outcome. Further studies are required to define the prognostic value of cytokine patterns, in terms of both GC treatment tailoring and timely initiation of IMV.

## 1. Introduction

In hospitalized patients with respiratory failure due to severe SARS-CoV-2 pneumonia or acute respiratory distress syndrome (ARDS), the progression from noninvasive mechanical ventilation (NIV) to invasive mechanical ventilation (IMV) is associated with significantly higher morbidity and mortality [1]. Risk factors for COVID-19 severity are still only partially understood, as also young non-comorbid patients may require IMV [2]. It is accepted that patients with severe COVID-19 have exhausted antiviral defenses and have an aberrant pulmonary and systemic inflammatory response, also referred to as a “cytokine storm,” that eventually becomes the leading cause of organ damage [3]. It has been demonstrated that the efficacy of corticosteroids on both the duration of mechanical ventilation and survival is higher than that of any other intervention [4]. Glucocorticoids are wide-spectrum anti-inflammatory agents that mainly exert their function via glucocorticoid receptor-alpha (GRα), mediated by nuclear factor-kB (NF-kB), along with other genomic and non-genomic pathways that ultimately lead to a reduced proinflammatory cytokine expression [5]. After the publication of the RECOVERY trial, several other studies and metanalyses confirmed the efficacy and safety of glucocorticoids (GCs) in COVID-19, although uncertainty remains about the best GC drug, as well as the dosing and duration of treatment [6]. In fact, one of the main unresolved issues is what underlies the failure of GC treatment in the subgroup of patients who require invasive respiratory support or do not survive. Patients with unresolving ARDS usually have both a persistent elevation in systemic inflammatory mediators (e.g., IL-1ß, IL-6, IL-8, TNF-α) and tissue resistance to GCs [7,8]. Indeed, GC treatment must be titrated on the severity of clinical conditions and inflammation, as we have recently reviewed [9]. Systemic cytokine levels observed in patients with SARS-CoV-2-induced ARDS have been suggested as a possible marker of disease severity and may also help to guide GC treatment [10]. Previous studies have reported on the main proinflammatory cytokine levels in patients with COVID-19 pneumonia of different severity, with discordant results. Single determinations of cytokine levels were performed in these studies at a predefined time-point [3,10,11]. We performed a single-center, observational study investigating the longitudinal profile of 27 cytokines in COVID-19 patients supported with NIV, in order to compare the response over time (days 7, 14, and 28) in those who deteriorated and progressed to IMV versus those who had successfully continued on NIV alone.

## 2. Methods

### 2.1. Study Population

Seventeen consecutive patients admitted to the Respiratory High Dependency Unit (RHDU) of the University Hospital of Trieste (Italy) due to SARS-CoV-2-induced ARDS [12,13] requiring NIV, between 12 March 2020 and 29 March 2020, were recruited. The study baseline was defined at the time of RHDU admission. All patients gave written informed consent before enrollment. The study was carried out in accordance with the Declaration of Helsinki, and it was approved by the referral Ethics Committee (#CEUR-2020-Os-052).

### 2.2. Study Design

Inclusion criteria were: SARS-CoV-2-positive (on nasopharyngeal swab or bronchial wash), age > 18 years, and a diagnosis of ARDS according to the Berlin definition [12]. Exclusion criteria were: heart failure as the main cause of acute respiratory failure, decompensated liver cirrhosis, immunosuppression (i.e., cancer on treatment, post–organ transplantation, HIV-positive, on immunosuppressant therapy), dialysis dependence, on long-term oxygen or home mechanical ventilation, idiopathic pulmonary fibrosis, neuromuscular disorders, dementia or a decompensated psychiatric disorder, severe neurodegenerative conditions, chronic steroid therapy, pregnancy, a do-not-resuscitate order, and the use of tocilizumab or other experimental treatments. All patients were given an 80 mg bolus of methylprednisolone at RHDU admission, followed by an infusion of 80 mg in 240 mL of normal saline solution at 10 mL/h for at least 8 days, until achieving either a PaO_2_:FiO_2_ > 350 mmHg or C-Reactive Protein (CRP) < 20 mg/L. The treatment was then de-escalated for 6 days until it was discontinued [14]. Furthermore, all patients received standard of care, including prophylactic anticoagulation with enoxaparin and awake pronation during mechanical ventilation, in line with the latest WHO guidelines for COVID-19 management [15].

Seriated blood samples were obtained at baseline and on days 7, 14, and 28 of the study. Serum samples were tested for a 27 cytokine panel by the Department of Advanced Translational Microbiology at the Institute for Maternal and Child Health-IRCCS “Burlo Garofolo” (Trieste, Italy), as already published [16]. The panel included: IL-1ß, interleukin-1 receptor antagonist (IL-1RA), interleukin-2 (IL-2), interleukin-4 (IL-4), interleukin-5 (IL-5), IL-6, interleukin-7 (IL-7), interleukin-8 (IL-8), interleukin-9 (IL-9), interleukin-10 (IL-10), interleukin-12 (IL-12), interleukin-13 (IL-13), interleukin-15 (IL-15), interleukin-17 (IL-17), eotaxin, fibroblast growth factor (FGF), granulocyte colony-stimulating factor (G-CSF), granulocyte–monocyte colony-stimulating factor (GM-CSF), interferon-gamma (IFN-γ), interferon gamma-induced protein 10 (IP-10), monocyte chemoattractant protein 1 (MCP-1), macrophage inflammatory protein-1-alpha (MIP-1α), platelet-derived growth factor-bb (PDGF-bb), macrophage inflammatory protein-1-beta (MIP-1β), chemokine ligand 5 (RANTES), tumor necrosis-alpha (TNF-α), and vascular endothelial growth factor (VEGF). The concentrations are expressed as pg/mL. C-reactive protein (CRP) levels (mg/L) were evaluated at the same time points. The remaining clinical, laboratory and outcome data were manually extracted from electronic medical records or charts.

### 2.3. Statistical Analysis

Data were described using absolute and relative frequencies (percentage) or position indices (mean) and relative dispersion indices (SD), as appropriate. The difference in numerical variables between groups was calculated by the Fisher’s exact test for categorical variables and with the Wilcoxon rank-sum test for all other variables. Sensitivity analyses were performed to account for the potential effect of baseline and demographic data on the study results.

## 3. Results

Five patients required IMV due to either a lack of improvement or a worsening of their respiratory status, as defined by PaO_2_:FiO_2_ being persistently <100 mmHg for >72 h or PaO_2_ < 55 mmHg despite maximal noninvasive support or signs of respiratory distress (respiratory rate above 30 breaths per minute, use of accessory respiratory muscles, agitation, sweating, hemodynamic instability), as assessed by the attending physician. From here on, these five patients will be referred to as the “IMV group” whilst the remaining 12 patients will be referred to as the “NIV group”.

There were five females (42%) in the NIV group vs. zero females (0%) in the IMV group, *p*-value = 0.24. The mean age was 60.4 ± 10.2 years in the NIV group vs. 69.0 ± 3.7 years in the IMV group, *p*-value = 0.07. NIV and IMV groups were also apparently balanced with regards to smoking history, BMI, major comorbidities, baseline PaO_2_/FiO_2,_ and laboratory data (Table 1) despite a low statistical power due to the small sample size.

**Figure 1:**
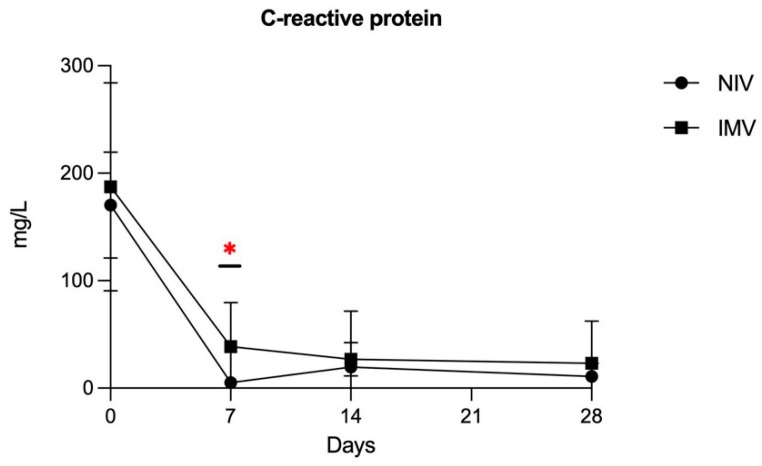
C-reactive protein time-course (mg/L) showing significantly higher levels in the IMV group only at day 7 (* *p*-value of Wilcoxon rank-sum test = 0.002).

No major complications occurred in the study groups. All 17 patients survived and were discharged without need for long-term oxygen therapy. The mean time from baseline to IMV initiation was 5 ± 2.9 days in the IMV group and the mean IMV duration was 10 ± 5.79 days. The mean duration of methylprednisolone infusion was 8.24 ± 1.21 days in the NIV group vs. 9.41 ± 1.92 days in the IMV group, *p*-value = 0.15. CRP levels were significantly higher in the IMV group on study day 7 (*p*-value = 0.002) but not on days 0 and 14 and 28 (Figure 1).

**Figure 2:**
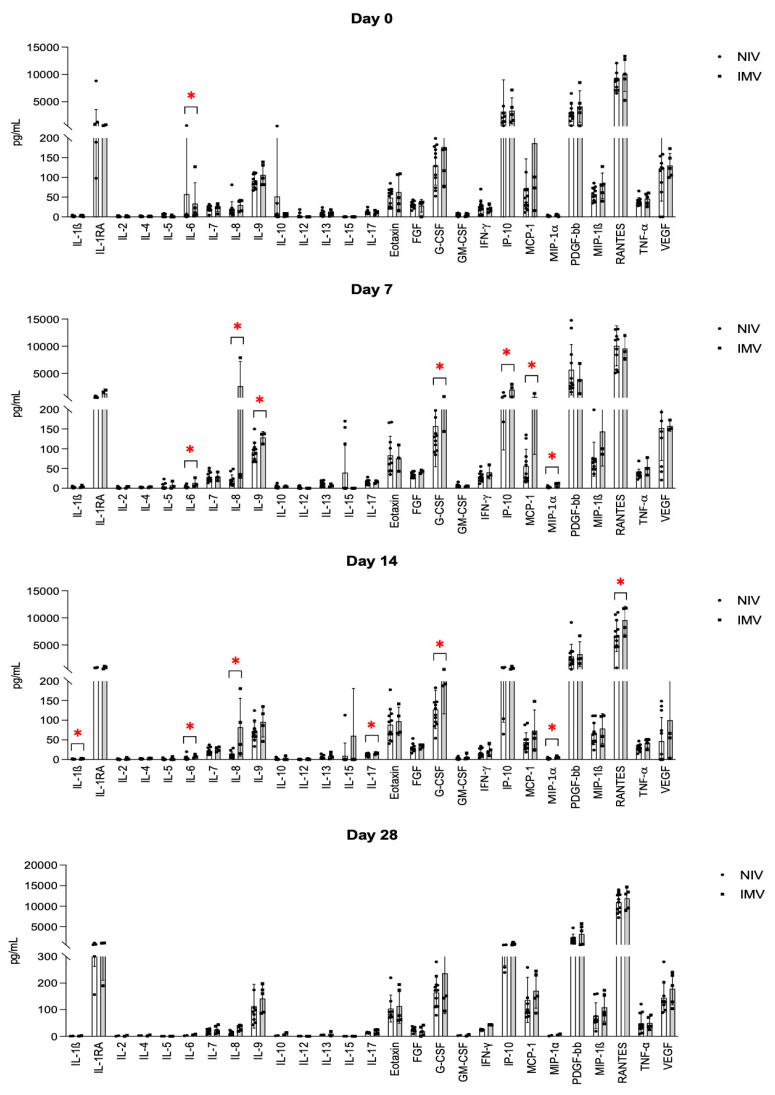
Serum cytokines levels (pg/mL) at different timepoints in NIV and IMV groups. IL-6 was significantly higher in the NIV group on day 0. IL-6, IL-8, IL-9, G-CSF, IP-10, MCP-1, and MIP-1α showed higher levels in the IMV group on day 7. IL-6, IL-8, IL-17, G-CSF, MIP-1α, and RANTES were higher in the same group on day 14. Note that IL-8 and G-CSF were higher at both days 7 and 14. * indicates statistical significance, *p*-value < 0.05.

IL-6 was significantly higher in the NIV vs. IMV group on day 0 (*p* = 0.035). Conversely, several cytokines showed a higher expression in the IMV group on day 7, i.e., IL-6 (*p* = 0.035), IL-8 (*p* = 0.052), IL-9 (*p* = 0.051), G-CSF (*p* = 0.052), IP-10 (*p* = 0.024), MCP-1 (*p* = 0.01), and MIP-1α (*p* = 0.024). On day 14, a significantly different expression between the IMV and NIV group was observed for IL-1β (*p* < 0.05), IL-6 (*p* = 0.038), IL-8 (*p* < 0.01), IL-17 (*p* = 0.043), G-CSF (*p* = 0.01), MIP-1α (*p* = 0.025), and RANTES (*p* = 0.045). No statistically significant differences were observed between the IMV and NIV groups on day 28. Figure 2 summarizes these data. Figure S1 shows the time-course of each cytokine levels at days 0, 7, 14, and 28.

## 4. Discussion

We observed higher IL-6 levels in the less severe (NIV) group at baseline, while several cytokines showed higher levels in the more severe (IMV) group on days 7 (IL-6, IL-8, IL-9, G-CSF, IP-10, MCP-1, MIP-1α) and 14 (IL-1β, IL-6, IL-8, IL-17, G-CSF, MIP-1α, RANTES). To note, IL-6 inverted its tendency between days 0 and 7, then IL-6, IL-8, and C-GSF remained persistently higher in the IMV group from day 7 to 14. This is in line with previous results from Leisman et al. [3]. C-reactive protein was also significantly more expressed in this group on day 7, despite the dramatic decline between days 0 and 7, which is attributable to the early administration of glucocorticoids. Indeed, CRP levels declined earlier than most other cytokines which followed the course of IL-6, as expected from the literature [17]. IL-8 showed the highest difference between groups at every time-point, consistent with previous literature data [10]. However, IL-8 also experienced the largest variations among cytokines within the IMV group, as its levels considerably raised by day 7 and remained high by day 14, suggesting that it might have the highest potential as a prognostic marker among the investigated cytokines. This result is concordant with the study from Li et al., who described that IL-8 levels were associated with in-hospital death among the most critical COVID-19 patients [18]. Other larger studies have been previously conducted with discordant results. In particular, Ghazavi et al. investigated the serum levels of interferon IFN-γ, IL-5, IL-8, Il-9, IL-17, and TGF-β in mild and severe COVID-19 patients vs. healthy controls, finding that only TGF-β levels were significantly higher in the COVID-19 groups [11].

In comparison with previous studies, we have longitudinally investigated the variations of a large 27-cytokine panel at different time-points (days 0, 7, 14, and 28) in patients supported with NIV. Furthermore, all patients in our study were treated with the same GC protocol that has already been shown to reduce mortality [14]. Following GC administration, a progressive and sustained reduction of all pro-inflammatory cytokines would have been expected [8,9]. Therefore, we speculate that patients in the IMV group experienced a certain degree of resistance to glucocorticoids that may explain the higher inflammation levels and a worse clinical evolution around day 7. Mechanisms underlying the intrinsic or acquired GC resistance include hereditary/epigenetic modifications or a reduced expression of GRα following a sustained exposure to inflammatory cytokines (e.g., IL-1β) and an increased activation of inflammation-related genes [19,20,21,22,23]. Although none of our study patients died, we hypothesize that higher GC doses and/or a longer duration of treatment might have been beneficial in achieving a better control of the inflammation and may have eventually led to a better outcome in the subset of patients who required IMV. Indeed, the advantages of a personalized approach, based on clinical response and inflammation markers, have been widely discussed and demonstrated [9].

IL6, IL-8, IL-9, IL-17, IP-10, MCP-1, G-CSF, MIP-1α, MIP-1β, and RANTES are pro-inflammatory cytokines stimulated by both pathogen presentation and tissue damage, and are involved at different levels in the activation of monocytes/macrophages and granulocytes in the context of the innate immune response. Elevated cytokine levels have been already reported in the acute phase of viral ARDS [10]; however, to the best of our knowledge, this is the first time in which persistent elevation has been correlated with a failure to improve and with the need for IMV.

We are aware that our study does have limitations that are mainly related to the small sample size, which might have masked an incomplete comparability of groups. In fact, statistical power was low with regards to all the variables of study, including baseline ones, and some outliers were noted among cytokine levels of the same patient at different time points. In our study, there were less females in the IMV group. Despite this, the difference was not statistically significant and it may not be surprising as the male gender has been associated with a higher intubation rate in COVID-19 ARDS; this should be considered as a limitation in light of the low sample size. Furthermore, cytokine expression is influenced by several external, genetic, and epigenetic factors other than GC treatment, that may hamper the pathophysiological interpretation of the average serum levels alone. Previous studies also demonstrated that there is substantial variability in the resulting plasma concentrations of individuals receiving the same GC dose [21]. As a small preliminary study, we performed a time-course analysis in distinct time points and outcome groups. Future research might focus on obtaining deeper information on the kinetics of the single cytokines over time.

Altogether, our findings indicate that high cytokine levels, i.e., IL-6, IL-8, and G-CSF, between day 7 and day 14 of hospitalization, in patients with SARS-CoV-2-induced ARDS on glucocorticoid treatment and NIV, correlate with NIV failure and with a need for IMV. In particular, L-8 levels increased dramatically from day 0 to day 7 and remained high until day 14 in the group of patients who required IMV during the same time frame, which may indicate glucocorticoid resistance. If confirmed by larger studies, these findings should prompt the need for the further investigation of a patient-tailored GC treatment protocol for COVID-19 and they may pave the way for future studies on the use of cytokine patterns as earlier prognostic tools to guide a timely initiation of IMV.

## Figures and Tables

**Table 1:** Distribution of the 17 study patients according to study group and baseline characteristics on day 0. Legend: SD, standard deviation; CRP, C-reactive protein; PaO_2_:FiO_2_, ratio of partial pressure of arterial oxygen (PaO_2_ in mmHg) to fractional inspired oxygen (FiO_2_%); COPD, chronic obstructive pulmonary disease; OSAS/OHS, obstructive sleep apnea syndrome/obesity-hypoventilation syndrome; LDH, lactate dehydrogenase; NIV, noninvasive ventilation group; IMV, invasive mechanical ventilation group. ° *p*-value of the Fisher’s exact test for dichotomous variables, Wilcoxon rank-sum test for numerical variables.

	NIV (*n* = 12)	IMV (*n* = 5)	*p*-Value °
Age, mean (SD)	60.4 (10.2)	69.0 (3.7)	0.07
Female sex, no. (%)	5 (42.0)	0 (0.0)	0.24
BMI ≥ 30 kg/m^2^, no. (%)	27.7 (6.5)	28.6 (2.8)	1.00
Ever smoker, no. (%)	3 (25.0)	3 (60.0)	0.28
Presence of major co-morbidities, no. (%)	8 (66.7)	5 (100.0)	0.26
Hypertension, no. (%)	3 (25.0)	1 (20.0)	1.00
Diabetes, no. (%)	4 (33.3)	2 (40.0)	1.00
Asthma/COPD, no. (%)	1 (8.3)	2 (40.0)	0.19
OSAS/OHS, no. (%)	1 (8.3)	2 (40.0)	0.19
Congestive heart failure, no. (%)	0 (0.0)	0 (0.0)	N/A
Ischemic cardiovascular disease, no. (%)	0 (0.0)	0 (0.0)	N/A
Chronic kidney disease, no. (%)	1 (8.3)	0 (0.0)	1.00
History of malignancy, no. (%)	1 (8.3)	1 (20.0)	0.51
PaO_2_:FiO_2_, mmHg, mean (SD)	161.0 (46.09)	112.9 (27.8)	0.07
CRP, mg/L, mean (SD)	170.4 (39.5)	187.4 (96.9)	0.83
D-dimer, ug/FEU/L, mean (SD)	714.3 (264.6)	580.0 (300.5)	0.30
LDH, U/L, mean (SD)	339.7 (74.4)	367.4 (81.9)	0.53
Lymphocyte count, mean (SD)	902.2 (291.0)	904.0 (381.2)	0.83

## Data Availability

Raw data are available upon request to the corresponding Author.

## Supplementary Materials

The following supporting information can be downloaded at: https://www.mdpi.com/article/10.3390/jcm11112951/s1, Figure S1. (A–C) Time-course of single cytokine levels (pg/mL) at days 0, 7, 14, 28. * indicates statistical significance, *p*-value < 0.05.

## Abbreviations

Glucocorticoids (GCs), noninvasive ventilation (NIV), invasive mechanical ventilation (IMV), acute respiratory distress syndrome (ARDS), body mass index (BMI), glucocorticoid receptor-alfa (GRα), nuclear factor-kB (NF-kB), interleukin 1-beta (IL-1ß), interleukin-1 receptor antagonist (IL-1RA), interleukin-2 (IL-2), interleukin-4 (IL-4), interleukin-5 (IL-5), IL-6, interleukin-7 (IL-7), interleukin-8 (IL-8), interleukin-9 (IL-9), interleukin-10 (IL-10), interleukin-12 (IL-12), interleukin-13 (IL-13), interleukin-15 (IL-15), interleukin-17 (IL-17), fibroblast growth factor (FGF), granulocyte-colony stimulating factor (G-CSF), granulocyte-monocyte-colony stimulating factor (GM-CSF), interferon-gamma (IFN-γ), Interferon gamma-induced protein 10 (IP-10), monocyte chemoattractant protein 1 (MCP-1), macrophage inflammatory protein-1-alpha (MIP-1α), platelet-derived growth factor-bb (PDGF-bb), macrophage inflammatory protein-1-beta (MIP-1ß), chemokine ligand 5 (RANTES), tumor necrosis-alpha (TNF-α), vascular endothelial growth factor (VEGF), C-reactive protein (CRP).
