# Supplementary material for: Cytokine Profiles as Potential Prognostic and Therapeutic Markers in SARS-CoV-2-Induced ARDS
Source: J Clin Med. 2022 May 24;11(11):2951. doi: 10.3390/jcm11112951 (PMC9180983; doi:10.3390/jcm11112951)
Supplement: Supplementary file 1 [file jcm-11-02951-s001.zip › jcm-1717210-supplementary.pdf]

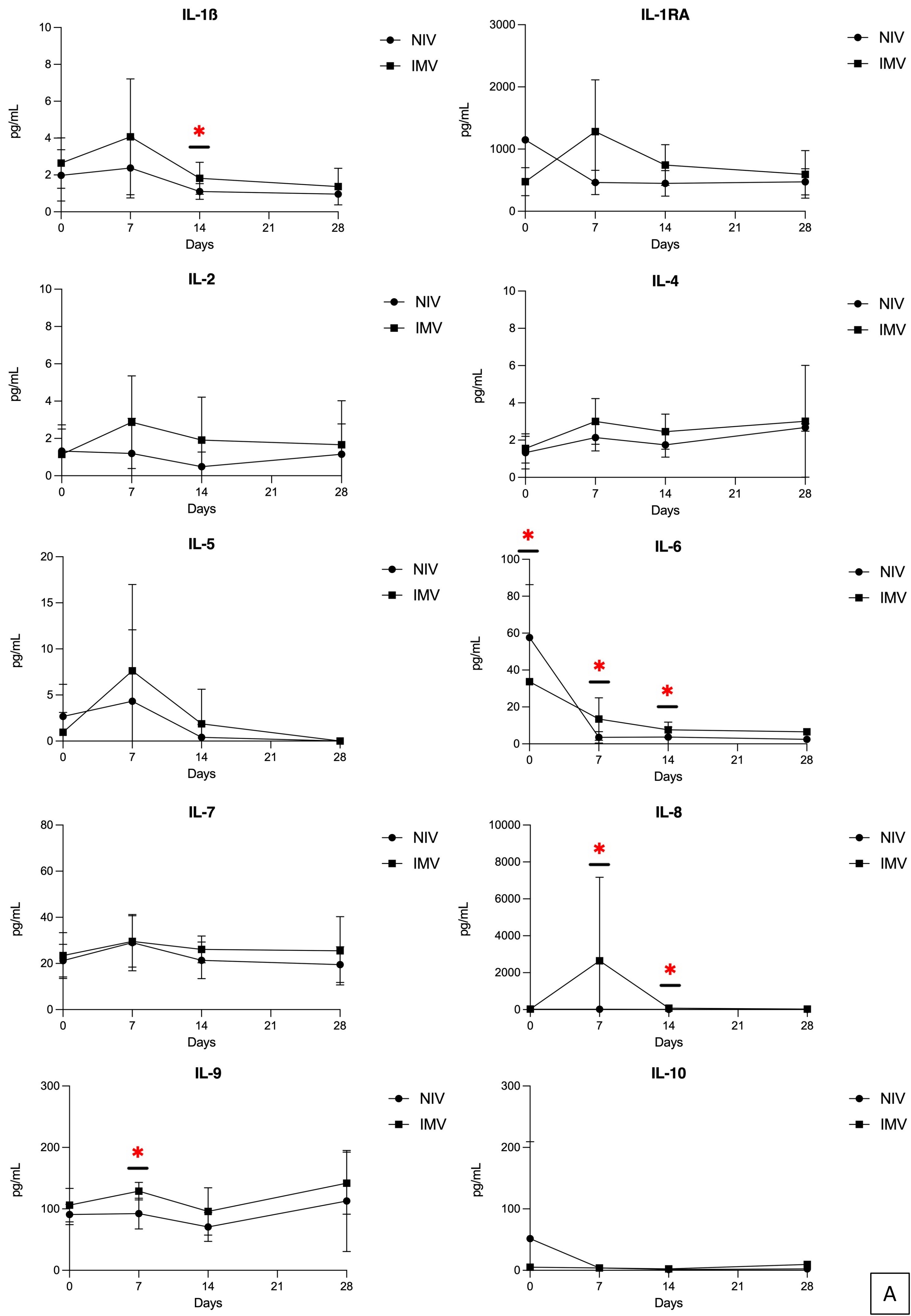
IL-1ß
IL-1RA
IL-2
IL-4
IL-5
IL-6
IL-7
IL-8
IL-9
IL-10
NIV
IMV
pg/mL
Days
A

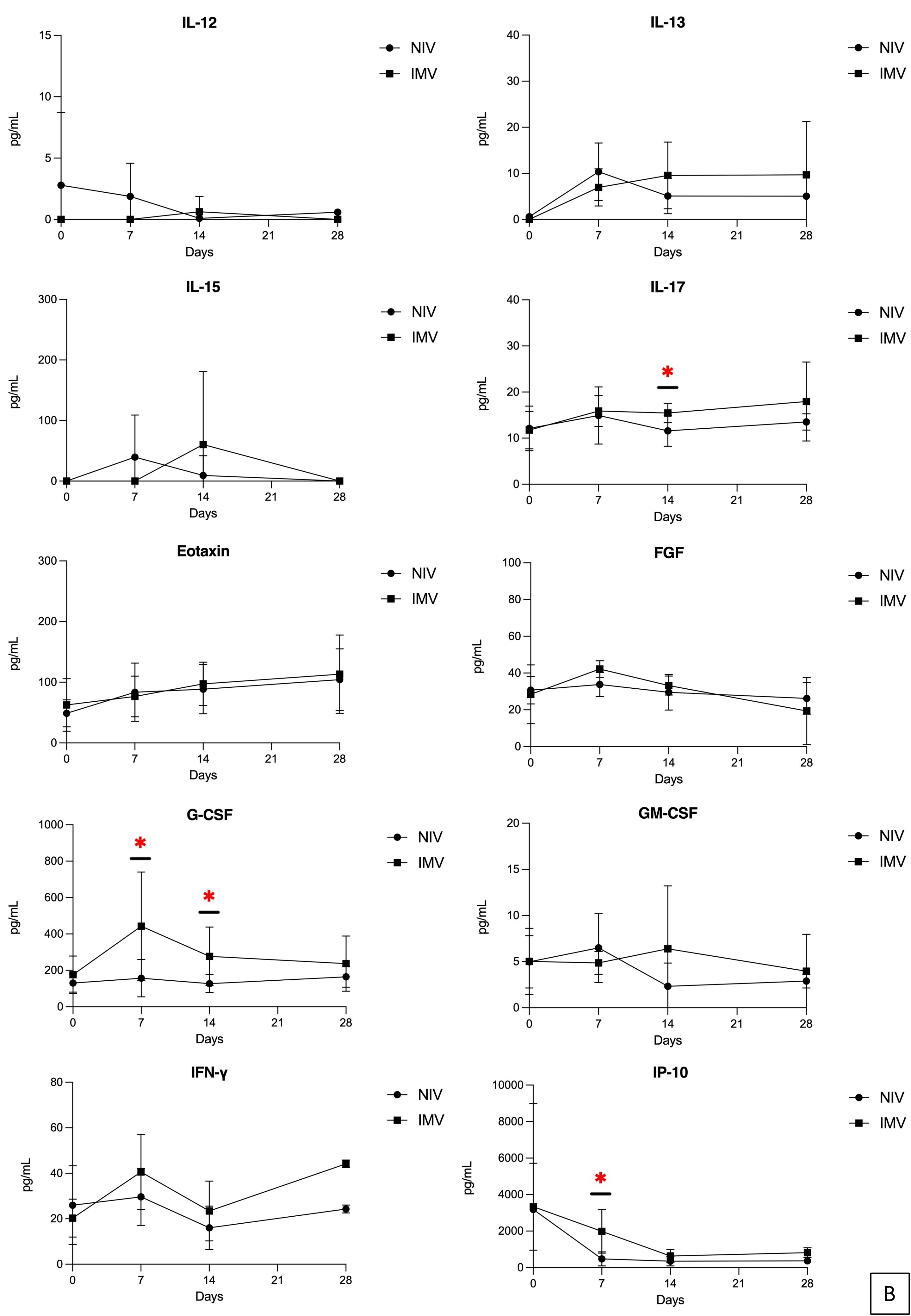
IL-12
IL-13
IL-15
IL-17
Eotaxin
FGF
G-CSF
GM-CSF
IFN-γ
IP-10
NIV
IMV
pg/mL
Days
0
7
14
21
28
*
B

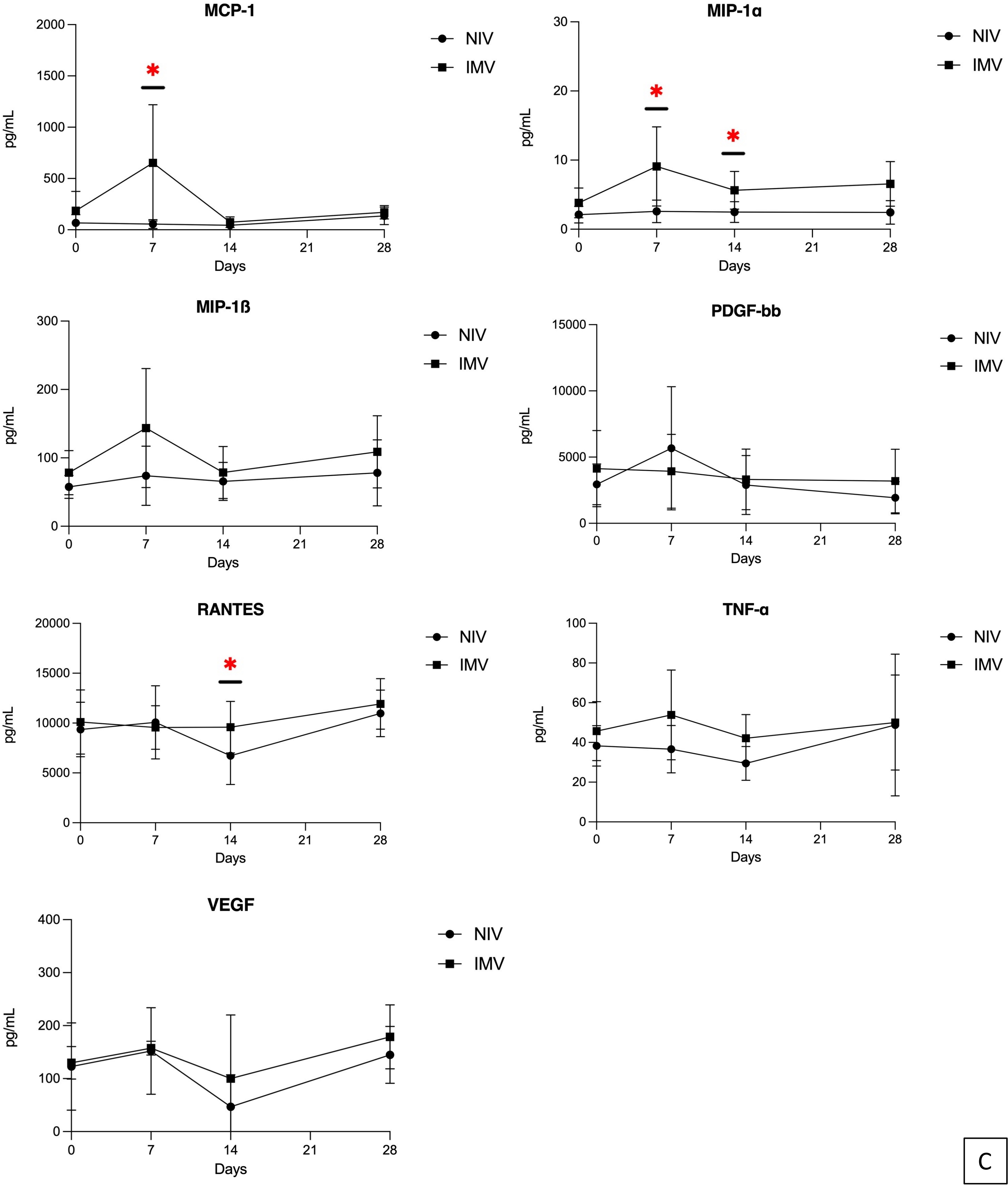

Figure S1 (**A-B-C**). Time-course of single cytokine levels (pg/mL) at days 0, 7, 14, 28. * indicates statistical significance, p-value < 0.05.
